# *Acrocalymmachuxiongense* sp. nov., a new species of Acrocalymmaceae (Pleosporales) on leaves of *Quercus*

**DOI:** 10.3897/BDJ.10.e89635

**Published:** 2022-08-19

**Authors:** Yu-Wei Liu, Xiang-Yu Zeng

**Affiliations:** 1 Department of Plant Pathology, College of Agriculture, Guizhou University, Guiyang 550025, China Department of Plant Pathology, College of Agriculture, Guizhou University Guiyang 550025 China

**Keywords:** morphology, new taxa, pathogen, phylogeny

## Abstract

**Background:**

In Huafo Mountain, Mouding, Yunnan Province, China, we found black protrusions on the leaf surface of *Quercus* plants. The collection which we identified as *Acrocalymmachuxiongense* sp. nov., a new species of Acrocalymmaceae (Pleosporales) is characterised by dome-shaped ascomata, bitunicate asci with pedicel furcate and an ocular chamber and hyaline, obovoid to ellipsoid, 1-septate ascospores.

**New information:**

Morphologically, this species is typical with obovoid to ellipsoid, larger ascospores than other known species in *Acrocalymma*. Phylogenetic analysis also showed that it represents a distinct clade, distant to any other species of *Acrocalymma*. Therefore, we introduce it as *Acrocalymmachuxiongense*, a new species of Acrocalymmaceae. This study is adding to the current situation where there are very few species and lack of teleomorph characteristics.

## Introduction

The genus *Acrocalymma* was introduced by Alcorn and Irwin (1987), to accommodate a root pathogen, *Acrocalymmamedicaginis*, on *Medicago* in Australia (Alcorn and Irwin 1987, Farr et al. 1998). The anamorph of *Acrocalymma* has cylindrical and hyaline conidia with a helmet-shaped mucilaginous appendage at each end (Alcorn and Irwin 1987, Zhang et al. 2012, Jayasiri et al. 2019). The teleomorph of *Acrocalymma* has ostiolate ascomata, 8-spored asci and fusiform ascospores, with hyaline sheath (Shoemaker et al. 1991). Zhang et al. (2012) described the second species, *A.aquatica* Huang Zhang & K.D. Hyde, from submerged wood in Thailand. The third species, *A.cycadis* Crous & R.G. Shivas, was introduced by Crous et al. (2014) and it differs from *A.medicaginis* and *A.aquatica* by its larger conidia. Trakunyingcharoen et al. (2014) revised the genus *Sphaerellopsis* and transferred *S.filum* (CBS 317.76) to *Acrocalymma*. They also proved that *Acrocalymma* and *Rhizopycnis* are congeneric, based on molecular and morphology results, therefore, transferred *Rhizopycnisvagum* to *Acrocalymma*. Likewise, *Massarinawalkeri* is synonymised under *Acrocalymma* (Trakunyingcharoen et al. 2014). Phylogenetically, the genus *Acrocalymma* represents an undefined lineage in the Pleosporales, so a new family Acrocalymmaceae was introduced by Trakunyingcharoen et al. (2014) to accommodate *Acrocalymma* as the type genus.

At present, *Acrocalymma* includes 11 species viz. *A.ampeli*, *A.aquaticum*, *A.bipolare*, *A.cycadis*, *A.fici*, *A.hongheense*, *A.medicaginis*, *A.pterocarpi*, *A.vagum*, *A.walkeri* and *A.yuxiense*. The major species are reported from terrestrial habitats (Hongsanan et al. 2020a, Hongsanan et al. 2020b, Tennakoon et al. 2021), while *A.aquaticum* and *A.bipolare* are freshwater species. *A.medicaginis* and *A.vagum* are reported as root pathogens on *Medicago* and *Cucumis*, respectively (Alcorn and Irwin 1987, Farr et al. 1998).

This study introduces a new species in *Acrocalymma* collected from Chuxiong, Yunnan, China, based on morphological description and phylogenetic analysis.

## Materials and methods

### Sample Collection and Isolation

Fresh fungal materials were collected from leaves of *Quercus* sp. in Huafo Mountain of Mouding County (Yunnan Province, China). The Mountain is located at 24˚09’–25˚40’N, 101˚18’–101˚51’E at an elevation of 1900–2588 m above sea level. The mountain ranges from north to south and is located on the windward side of the southeast airstream, which is warm, humid and rainy and the air humidity is high. This area also has an annual average rainfall of 1029 mm and an annual average temperature of 12.1˚C–13.5˚C (Zhang et al. 2012). The collected specimens were brought to the laboratory in paper envelopes. Samples were examined with a compound light microscope (Zeiss Scope 5). Healthy leaf tissues and the margins of diseased tissues of each leaf spot were cut into 6 mm square sections and surface-sterilised as follows. These sections were initially soaked in 0.5% sodium hypochlorite for 2 minutes, then 1 minute in sterile distilled water, 2 minutes in 75% ethanol and, finally, 1 minute in sterile distilled water. The sterilised fragments were then plated on potato dextrose agar and incubated at 25°C for 6-8 days or until mycelia growing from the leaf fragments were observed (Yu et al. 2022). The holotype was deposited at the Herbarium of IFRD (International Fungal Research & Development Centre; Institute of Highland Forest Science, Chinese Academy of Forestry, Kunming, China). The ex-type living culture was deposited at the Culture Collection of the Herbarium of IFRD (IFRDCC) (Li et al. 2022). The MycoBank number was registered (Crous et al. 2014).

### Morphological Observations

Photographs of the ascomata were taken using a stereomicroscope (Keyence VHX-7000 digital microscope). Observations and photomicrographs were made from material mounted in lactic acid (60%) using a compound light microscope (Zeiss Scope 5) equipped with an AxioCam 208 colour camera with interference contrast optics. All measurements were made with ZEN2 (blue edition) and images used for figures were processed with Adobe Photoshop version 2022 software.

### DNA Extraction, PCR Amplififications and Sequencing

Fungal isolates were grown on PDA for 20 days at 25°C in the dark. DNA was extracted from the pure culture with a Biospin Fungus Genomic DNA Extraction Kit (BioFlux, China) according to the manufacturer’s instructions (Hangzhou, P.R. China). The internal transcribed spacer (ITS), along with the 5.8S ribosomal rDNA, was amplified with the primer pair ITS1 and ITS4 (White et al. 1990). The partial large subunit (LSU) ribosomal rDNA was amplified with the primer pair LR0R and LR5 (Vilgalys and Hester 1990, Rehner and Samuels 1994). The amplification reactions were carried out with the following protocol: 20 μl reaction volume containing 1 µl of DNA template, 2 µl of each forward and reverse primers, 17 µl of GoldenStar T6 Super PCR Mix (1.1×). The PCR conditions were: an initial denaturation step of 5 min at 95°C, followed by 35 cycles of 30 s at 95°C, 50 s at 55°C (ITS) and 55°C (LSU) and 90 s at 72°C and a final elongation step of 10 min at 72°C (Wanasinghe et al. 2020). PCR amplification products were assayed via electrophoresis in 1% agarose. The PCR products were sent to Tsingke Biotechnology Co., Ltd., Beijing, China. The nucleotide sequence data acquired was deposited in GenBank (Table 1).

### Phylogenetic Analyses

Sequences of each gene generated from forward and reverse primers were assembled with BioEdit version 7.2.5 (Hall 1999) to obtain consensus sequences. Related sequences were selected and downloaded from GenBank. Each gene dataset was aligned separately by MAFFT version 7.187 (Katoh and Standley 2013) and manually aligned where necessary. Phylogenetic trees, based on LSU and ITS individual datasets as well as a concatenated dataset (LSU and ITS), were analysed using Maximum Likelihood (ML) and Bayesian Inference at the CIPRES web portal (Miller et al. 2010). The ML analysis was carried out using the RAxML‐HPC BlackBox tool (Stamatakis 2014). One thousand non-parametric bootstrap iterations were used with a general time reversible (GTR) model and a discrete gamma distribution, plus estimating the proportion of invariable sites (Stamatakis et al. 2008). Bayesian Inference was undertaken using the MrBayes on XSEDE tool (Ronquist et al. 2012), based on the nucleotide substitution models determined by the Bayesian information criterion using the jModelTest2 on XSEDE tool (Darriba et al. 2012) at the CIPRES web portal. The Markov Chain Monte Carlo algorithm of four chains started from a random tree topology with two parallel runs. Trees were sampled every 1000 generations and the run was stopped automatically when the average standard deviation of split frequencies fell below 0.01. A 50% majority rule consensus tree was summarised after discarding the first 25% of samples. The resulting trees were visualised in FigTree version 1.4.3.

## Taxon treatments

### Acrocalymma chuxiongense

Y. W. Liu & X. Y. Zeng sp. nov.

C9A75F0A-E0D9-56CA-94F3-B5F6D1C6A8B0

844399

#### Materials

- **Type status:** Holotype. **Occurrence:** catalogNumber: IFRD9449; recordedBy: Liu Yu-Wei; occurrenceID: living culture IFRDCC3104; **Taxon:** scientificName: *Acrocalymmachuxiongense*; kingdom: Fungi; class: Dothideomycetes; order: Pleosporales; family: Acrocalymmaceae; genus: Acrocalymma; **Location:** country: China; stateProvince: Yunnan; county: Mouding; locality: Huafo Mountain; locationRemarks: Yunnan, Mouding, Huafo Mountain, 2021.10.7, Liu Yu wei; verbatimCoordinates: 25°19'8"N 101°25'7"E; **Identification:** identifiedBy: Yu-Wei Liu; dateIdentified: 2022; **Record Level:** language: en

#### Description

Habitat terrestrial, epiphytic with dome-shaped black protrusions on living leaves of *Quercus* sp. **Teleomorph**: Ascomata 50–80 µm high, 270–320 µm in diam. (average = 70 × 280 µm, n = 10), dark brown, gregarious, erumpent to nearly superficial, visible as numerous, raised, dome-shaped areas on host surface, globose, uni-loculate, glabrous with rough walls, coriaceous. Peridium 10–30 µm wide, of unequal thickness, composed of dark brown to black cells, arranged in *textura angularis*. Asci 130–170 × 40–45 µm, (average = 150 × 40 µm, n = 20), 8-spored, bitunicate, pedicel furcate, apically rounded with an ocular chamber. Ascospores 35–45 × 18–20 µm, (average = 40 × 20 µm, n = 40), overlapping, bi-seriate, hyaline, obovoid to ellipsoid, 1-septate, constricted at the septum, with conically rounded ends, upper cell larger than lower cell, smooth-walled (Fig. 2). **Anamorph**: Undetermined.

**Culture Characteristics**: Colonies grew on PDA at 25^0^C in the dark and reached 4 cm in diam., within 14 days, dense, circular, slightly raised in the middle, entire margin off-white to grey in surface view.

#### Etymology

The specific epithet reflects Chuxiong, from where the specimen was collected.

#### Notes

**Figure 1.**
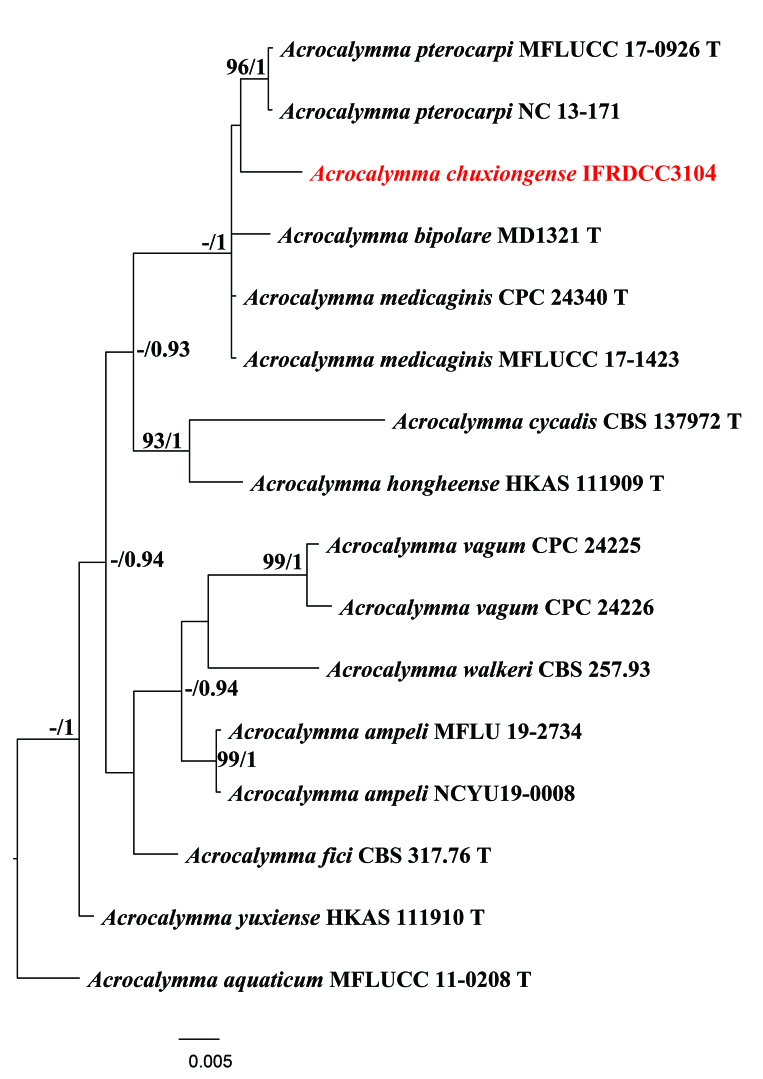
Bayesian Inference (BI) phylogenetic tree, based on a combined dataset of partial LSU and ITS sequence data. Bootstrap support values for ML equal to or greater than 50%, Bayesian posterior probabilities equal to or greater than 0.9 are shown as ML/BI above the nodes. The new isolates are in red. The scale bar represents the expected number of nucleotide substitutions per site. The tree was rooted with *Acrocalymmaaquaticum* (MFLUCC 11-0208).

**Figure 2.**
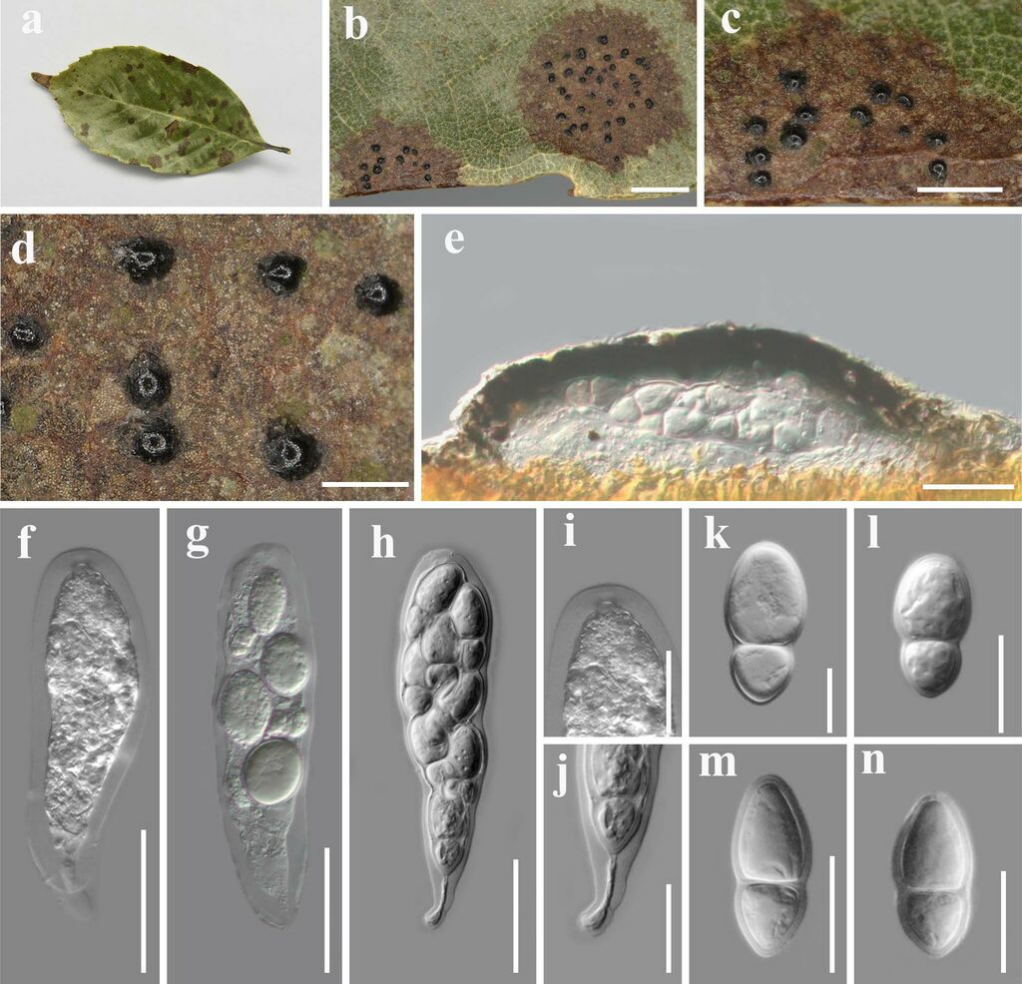
*Acrocalymmachuxiongense* (IFRD9449). **a–d** Ascomata on living leaves of *Quercus* sp.; **e** Vertical sections through a ascoma; **f–h** Asci; **i** Ocular chamber; **j** Pedicel; **k–n** Ascospores. Scale bars: b = 2000 µm, c = 1000 µm, d = 500 µm, e–h = 50 µm, i–n = 25 µm.

The teleomorph of *Acrocalymmachuxiongense* was similar to *A.pterocarpi*, but can be distinguished by the shape and size of ascospores. Our new collection has a larger ascospore (35–45 × 18–20 µm) than *Acrocalymmapterocarpi* (17–21 × 3–5 µm) (Jayasiri et al. 2019). The shape of ascospores of the new collection is obovoid to ellipsoid, while *Acrocalymmapterocarpi* has fusiform ascospores. Additionally, the upper cells of our new collection’s ascospores are larger than the lower cells, while the upper and lower cells of *A.pterocarpi* are similar in size. Phylogenetic analysis, based on ITS and LSU sequence data, showed that *Acrocalymmachuxiongense* forms a distinct lineage sister to *A.pterocarpi* (Fig. 1). The GenBank accession number for TEF of our new isolate is ON604684. A comparison of the ITS and LSU nucleotides of *A.chuxiongense* and *A.medicaginis* (the type) reveals 10 (2%) and 3 (1%) nucleotide differences. Comparing the ITS and LSU nucleotides of *A.chuxiongense* and *A.pterocarpi*, there are 11 (3%) and 5 (1%) nucleotide differences, respectively.

## Analysis

### Phylogenetic Analyses

The alignment comprised 16 strains with 1322 total characters including gaps. The best nucleotide substitution model for LSU and ITS is K80+I and TIM2ef+G, respectively. The RAxML analysis of the combined dataset yielded a best scoring tree with a final ML optimisation likelihood value of -2834.090234. Estimated base frequencies are as follows: A = 0.240753, C = 0.220941, G = 0.279900, T = 0.258406; substitution rates AC = 3.347637, AG = 2.128594, AT = 3.236536, CG = 0.316367, CT = 10.359429, GT = 1.000000; proportion of invariable sites I = 0.752805; gamma distribution shape parameter α = 0.633740. The Bayesian analysis ran (92000) generations before the average standard deviation for split frequencies reached (0.008966). The analysis generated (1842) trees, from which 1382 were sampled after 46 of the trees were discarded as burn-in. Our new collection forms a distinct clade, distant to any other species in *Acrocalymma* (Fig. 1).

## Discussion

In this study, we introduced a new species, *Acrocalymmachuxiongense*, from living leaves of *Quercus*, based on phylogenetic analysis and morphological comparisons.

*Acrocalymma* species are able to produce pycnidia in culture easily (Trakunyingcharoen et al. 2014). It can be seen from Table 2 and Table 3 that, amongst the 11 species of *Acrocalymma*, only *A.pterocarpi*, *A.hongheense* and *A.walkeri* have teleomorph characteristics and the others have only anamorph characteristics. However, we failed to obtain the anamorph of *Acrocalymmachuxiongense* under similar conditions. In addition, the ostiole, hamathecium and sheath were not observed in our collection. At the same time, the species of *Acrocalymma* are mainly distinguished by the size of conidia. It can be seen from Table 2 that the ascospores of our new isolate are the largest compared with *A.pterocarpi*, *A.hongheense* and *A.walkeri*. The ascospores of our new isolate are obovoid to ellipsoid and the other three are fusiform.

All species of *Acrocalymma* have sequence data in GenBank, but most have only ITS and LSU sequences and lack SSU and TEF sequences. On the other hand, sequence data of TEF were not used in the phylogenetic analyses due to lack of relevant data. Enriching the sequence data will provide more comprehensive phylogenetic relationships of the genus.

So far, only 11 species of *Acrocalymma* have been reported, indicating that *Acrocalymma* has great research potential. The future investigations of *Acrocalymma* will reveal more undiscovered species.

## Supplementary Material

XML Treatment for Acrocalymma chuxiongense

## Figures and Tables

**Table 1.** Taxa used in the phylogenetic analysis of Acrocalymmaceae and their corresponding GenBank numbers. The newly generated sequences are indicated in bold. NA: Sequence data not available in GenBank. T = ex-type strain.

**Species**	**Strain no.**	**GenBank accession no.**		**Reference**
		**ITS**	**LSU**	
*Acrocalymmaampeli*	MFLU 19-2734	MW063150	MW063211	Tennakoon et al. (2021)
*Acrocalymmaampeli*	NCYU19-0008	MW063151	MW063212	Tennakoon et al. (2021)
*Acrocalymmaaquaticum*	MFLUCC 11-0208 T	JX276951	JX276952	Zhang et al. (2012)
*Acrocalymmabipolare*	MD1321 T	NA	MN913734	Dong et al. (2020)
*Acrocalymmacycadis*	CBS 137972 T	KJ869124	KJ869181	Crous et al. (2014)
***Acrocalymmachuxiongense***	**IFRDCC3104**	**ON595715**	**ON596248**	**This study**
*Acrocalymmafici*	CBS 317.76 T	KP170619	KP170712	Trakunyingcharoen et al. (2014)
*Acrocalymmahongheense*	HKAS 111909 T	MW424761	MW424775	Mortimer et al. (2021)
*Acrocalymmamedicaginis*	CPC 24340 T	KP170620	KP170713	Trakunyingcharoen et al. (2014)
*Acrocalymmamedicaginis*	MFLUCC 17-1423	MT214338	MT214432	Mapook et al. (2020)
*Acrocalymmapterocarpi*	MFLUCC 17-0926 T	MK347732	MK347949	Jayasiri et al. (2019)
*Acrocalymmapterocarpi*	NC 13-171	LC517880	LC517881	Unpublished
*Acrocalymmavagum*	CPC 24226	KP170636	NA	Trakunyingcharoen et al. (2014)
*Acrocalymmavagum*	CPC 24225	KP170635	NA	Trakunyingcharoen et al. (2014)
*Acrocalymmawalkeri*	CBS 257.93	MH862398	FJ795454	[1] Zhang et al. (2009) [2] Vu et al. (2019)
*Acrocalymmayuxiense*	HKAS 111910 T	NA	MW424778	Mortimer et al. (2021)

**Table 2.** Comparison of teleomorph of *Acrocalymma* sp.

Species	Ascomata	Asci	Ascospores	Locality	Reference
*Acrocalymmachuxiongense*	50–80 µm high, 270–320 µm in diam., gregarious, dark brown.	130–170 × 40–45 µm	35–45 × 18–20 µm, hyaline, obovoid to ellipsoid, 1-septate, upper cell larger than lower cell.	Yunnan	This study
*Acrocalymmahongheense*	180–220 µm high, 160–200 µm diam., gregarious, dark brown, ostiolate.	100–140 × 15–22 µm	25–35 × 9.5–11 µm, hyaline, 1-septate, fusiform, with a sheath, the expansion near the septate.	Yunnan	Mortimer et al. (2021)
*Acrocalymmapterocarpi*	140–150 µm high, 130–145 µm diam., scattered, black, without ostiole.	65–75 × 7–12 µm	17–21 × 3–5 µm, hyaline, fusiform, 1-3 septate, guttulate, sheath present in immature stage.	Thailand	Jayasiri et al. (2019)
*Acrocalymmawalkeri*	160–180(225) µm wide, 160–180(225) µm high, covered with light grey hairs.	50–80 × 8–11µm	19–22 × 4.5–5.5 µm, pale reddish-brown, 3-septate, fusiform, with a sheath.	Australia	Shoemaker et al. (1991)

**Table 3.** Comparison of anamorph of *Acrocalymma* sp.

Species	Conidia				Locality	Reference
	Size	Color	Shape	Apex and base		
*Acrocalymmahongheense*	20–35 × 7–9 µm	hyaline	subcylindrical	obtusely rounded and with mucoid ooze at the apex, protuberant and with a rounded hilum at base.	Yunnan	Mortimer et al. (2021)
*Acrocalymmayuxiense*	15–21 × 4–5 µm	hyaline	subcylindrical	obtusely rounded at apex and base, guttulate.	Yunnan	Mortimer et al. (2021)
*Acrocalymmafici*	(12–)13–15(–16) × 2.5(–3) µm	hyaline	cylindrical	cylindrical with subobtuse apex, acutely tapered at base to a small flattened central scar, with flaring mucoid apical appendage, visible in water mounts.	India	Trakunyingcharoen et al. (2014)
*Acrocalymmamedicaginis*	(11–)13–15(–16) × (3.5–)4 µm	hyaline	subcylindrical	apex obtuse, tapering at base to truncate hilum, ends with mucoid caps.	Australia	Trakunyingcharoen et al. (2014)
*Acrocalymmavagum*	(16–)18–25(–28)×(4.0–)4.5–6.0(–6.9) µm	hyaline to brown	cylindrical to fusiform	apex rounded, base obtuse or tapering abruptly to a truncate base, guttulate.	Spain, USA	Farr et al. (1998)
*Acrocalymmaampeli*	17–19 × 5.5–6.5 µm	hyaline	cylindrical to fusoid	apex obtuse, unicellular, with flaring mucoid apical appendage at lower end, visible in water mounts.	Taiwan	Tennakoon et al. (2021)
*Acrocalymmaaquatica*	12–17 × 3–4 µm	hyaline	cylindrical to fusoid	truncate at the base and becoming a little narrower at apex with a mucilaginous helmet-shaped appendage.	Thailand	Zhang et al. (2012)
*Acrocalymmabipolare*	9–12 × 3–5 µm	hyaline	cylindrical to fusiform	with rounded apex and slightly narrow, truncate base, with mucoid polar appendages that are filled with oil droplets, appendages elongate in water to form filaments.	Egypt	Dong et al. (2020)
*Acrocalymmacycadis*	(25–)28–32(–35) × (4–)5 µm	hyaline	subcylindrical	apex obtusely rounded, hilum truncate, guttulate.	Australia	Crous et al. (2014)
